# Weight self-perception and weight loss attempts in Chinese cardiovascular patients and non-cardiovascular patients: evidence from a population-based study

**DOI:** 10.1186/s12889-023-15380-w

**Published:** 2023-04-19

**Authors:** Qingyuan Gao, Ruotong Li, Zhiteng Chen, Wenyao Yin, Guanghong Liao, Haifeng Zhang, Jingfeng Wang, Yangxin Chen

**Affiliations:** 1 Department of Cardiology, Sun Yat-sen Memorial Hospital, Sun Yat-sen University, Guangzhou, Guangdong 510120 People’s Republic of China; 2 Laboratory of Cardiac Electrophysiology and Arrhythmia in Guangdong Province, Guangzhou, Guangdong 510120 People’s Republic of China; 3 Department of Epidemiology and Health Statistics, Xiangya School of Public Health, Central South University, Changsha, 410078 People’s Republic of China

**Keywords:** Weight self-perception, Weight misperception, Cardiovascular patients, Obesity, Weight loss

## Abstract

**Background:**

Weight loss is a significant improvement for individuals with overweight or obesity, especially for cardiovascular patients. The driving effects of weight self-perception and attempts to lose weight are vital in weight management, yet weight misperception is a direct culprit for the undesirability of weight control and obesity prevention. This study aimed to investigate weight self-perception and misperception and weight loss attempts in Chinese adults, especially among cardiovascular and non-cardiovascular patients.

**Methods:**

We collected data from China HeartRescue Global Evaluation Baseline Household Survey 2015. Questionnaires were used to assess self-reported weight and cardiovascular patients. We used kappa statistics to check the consistency between weight self-perception and BMI. Logistic regression models were fitted to identify risk factors associated with weight misperception.

**Results:**

A total of 2690 participants were enrolled in the household survey, while 157 respondents were cardiovascular patients. According to questionnaire results, 43.3% of cardiovascular patients thought they were overweight and obese, while the percentage is 35.3% among non-cardiovascular patients. Kappa statistics indicated higher consistency of self-reported weight and actual weight among cardiovascular patients. Multivariate analysis showed weight misperception was significantly associated with gender, education level, and actual BMI. Lastly, 34.5% of non-cardiovascular patients and 35.0% of cardiovascular patients were trying to lose weight or keep weight. The majority of these people adopted combined strategies of controlling diet and exercise to lose or maintain weight.

**Conclusions:**

Weight misperception was highly prevalent among cardiovascular or non-cardiovascular patients. Obese respondents, women, and individuals with lower education levels were more vulnerable to make weight misperception. However, no difference in the purpose of weight loss attempts was indicated among cardiovascular and non-cardiovascular patients.

## Background

Overweight and obesity are defined as abnormal or excessive fat accumulation, which is a considerable global health challenge that urgently needs to be resolved. Overweight and obesity not only lead to the onset of cardiovascular disease (CVD) for non-CVD individuals but also cause CVD development and even major adverse cardiovascular outcomes among CVD patients, independently of other cardiovascular risk factors [1–3]. There is growing evidence demonstrating the improvement of other CVD risk factors from moderate weight loss, such as lowering blood pressure, decreasing blood lipid, and ameliorating insulin resistance [4, 5]. Besides, the benefits of weight loss as a secondary prevention measure are also prominent, which decrease the risk of recurrent cardiovascular events among individuals with CVD [6]. More notably, CVD patients are more urgent for weight management. EUROASPIRE IV and V studies showed that over 80% of participants with coronary heart disease failed to achieve the weight loss target [7]. Therefore, weight management is crucial and beneficial for individuals with overweight or obesity, particularly for CVD patients.

Weight self-perception is considered as the degree of concordance between self-reported weight and actual weight [8], which may be reflected in some behavioral and emotional responses that indicate weight loss attempts. The self-perception of body weight and attempts to lose weight are vital for weight management and maintenance. And then, there is a growing body of studies that demonstrated weight-loss strategies are connected with successful weight loss in the obese population in the United States and China, yet weight misperception is a direct culprit for the undesirability of weight control and obesity prevention [9–11]. However, the previous literature mainly emphasized weight self-perception among the general population with different gender, age, ethnicity, BMI, education, and socioeconomic status [12–14]. Systematic research about weight self-perception and weight loss attempts among Chinese CVD patients is not available.

Thus, assessing the current situation of weight self-perception and weight loss attempts in different Chinese populations remains highly needed, especially in Chinese CVD patients. Based on comparable data from the China HeartRescue Global Evaluation Baseline Household Survey, we aimed to estimate weight self-perception, misperception, and weight loss attempts in Chinese adults. We also focused on weight self-perception and weight loss differences between CVD and non-CVD patients.

## Methods

### Studying design and population

Data were derived from China HeartRescue Global Evaluation Baseline Household Survey 2015 [15]. The public dataset includes the results of a baseline household survey conducted in Beijing and Shanghai, China. Data were collected from 1500 individuals ages 18 or older in each city, for a total of 3000 respondents. Information was collected from respondents through computer-assisted personal interviews (CAPI). Data were collected about demographics, health history and status, health behaviors, health care use, and knowledge, attitudes, and practices regarding CVD, risk factors, and CVD care.

In this study, a total of 2796 individuals with informed consent were included from the data source. We excluded participants with outliers in age (*n* = 4) and participants with missing information or being refused to answer in study variables (*n* = 102), giving a total of 2690 respondents for the final analysis.

### Sampling method

Stratified random sampling was used to select households in pilot cities of China HeartRescue Global Evaluation Baseline Household Survey (including Beijing and Shanghai). Two counties and districts were selected from each of the two cities. Within each county/district, streets or townships were stratified into four levels (distant, moderate-distant, nearby, and neighbored) according to the distance between the community and the survey hospital. One street or township was selected per stratum. Within each street or township, four residential committees or villages were selected by probability proportional to size, and 48 residents ages 18 and older were selected per committee/village using simple random sampling until an age-sex quota was met that matched the age-sex distribution of the county/district [15].

### Study variables

Self-reported body weight was assessed with the question, ‘How do you feel about your current weight?’, corresponding option value, ‘(1) Slim; (2) Normal; (3) Overweight; (4) Obese.’ For analysis purposes, these four response options were matched with the body mass index (BMI) categories: (1) Underweight; (2) Normal; (3) Overweight; (4) Obesity [16]. Whether taking measures to control weight or not and specific measures to lose or maintain the current weight of respondents were included for analysis. The accordance between actual weight status and self-reported weight status was analyzed as weight self-perception and reported as: (1) correct perception: when the self-reported weight status is equal to actual weight status; (2) misperception: when the self-reported weight status is lower or higher than the actual weight status.

Actual weight was estimated by BMI, which was calculated by the original current weight and height obtained from the questionnaire. BMI value was divided into four groups—underweight (< 18.5 kg/m^2^), normal (18.5–23.9 kg/m^2^), overweight (24–27.9 kg/m^2^), and obesity (> 28.0 kg/m^2^).

Cardiovascular patients were defined by the question, ‘Have you ever been diagnosed with any of these conditions? (1) Heart disease; (2) Angina; (chest pain from heart disease); (3) Heart attack; (4) Heart failure; (5) Myocardial infarction (MI); (6) Stroke (cerebrovascular accident or incident); (7) ST-segment elevated myocardial infarction (STEMI); (8) Cardiac arrest (cardiopulmonary arrest, circulatory arrest, or sudden death); (9) Congestive heart failure; (10) Atrial fibrillation.’

Sociodemographic factors of respondents include gender, age, marital status, and education level.

### Statistical analysis

Descriptive data were used for demographic data, BMI group, weight self-perception, the purpose of controlling weight, and measures taken to control weight. Rao-Scott *χ*^*2*^ test was used to test weight self-perception between CVD and non-CVD patients. Kappa statistics were estimated to check consistency between self-perception of weight and actual BMI separately for CVD and non-CVD patients. A kappa statistic of 1 indicates perfect agreement between the two observers, while a kappa statistic of 0 indicates that agreement could have been due to chance. Multivariate logistic regression models were fitted to identify factors associated with a misperception of the body weight and evaluate whether it was associated with CVD. Data were analyzed using IBM SPSS Statistics version 25.0.

## Results

### Characteristics of respondents in the household survey

There were 2690 valid responses out of 2796 in the household survey, while 157 respondents were defined as cardiovascular patients. Compared to non-CVD patients, the proportion of females was higher, and the age structure was older in CVD patients. More individuals were married in CVD patients, while non-CVD patients had advanced degrees. The percentages of respondents with overweight and obesity were substantially higher in CVD patients (Table 1).

**Table 1:** Characteristic of respondents in the household survey in China

	Non-CVD^a^ patients (*n* = 2533)		CVD patients (*n* = 157)		*P*
	***n***	***%***	***n***	***%***	
Gender					0.012
Male	1261	49.8	62	39.5	
Female	1272	50.2	95	60.5	
Age groups					< 0.001
18-29	674	26.6	1	0.6	
30-39	556	22.0	7	4.5	
40-49	453	17.9	11	7.0	
50-59	441	17.4	28	17.8	
60-69	287	11.3	69	43.9	
≥ 70	122	4.8	41	26.1	
Marital status					< 0.001
Married	1901	75.0	152	96.8	
Other marital status^b^	632	25.0	5	3.2	
Education level					< 0.001
Less than secondary school	578	22.8	88	56.1	
High school	562	22.2	37	23.6	
College	570	22.5	15	9.6	
University or higher	784	31.0	14	8.9	
Missing	39	1.5	3	1.9	
BMI group^c^					0.01
Underweight	107	4.2	4	2.5	
Normal	1428	56.4	70	44.6	
Overweight	764	30.2	64	40.8	
Obesity	234	9.2	19	12.1	

Values are presented with n and %, unless otherwise indicated. ***χ***^**2**^ tests were used for categorical variables

^a^*CVD* Cardiovascular diseases

^b^Other marital status includes divorced, widowed or unmarried respondents

^c^Body mass index was divided into four groups—underweight (< 18.5 kg/m^2^), normal (18.5–23.9 kg/m^2^), overweight (24–27.9 kg/m^2^), and obesity (> 28.0 kg/m^2^)

### Self-reported weight status and weight self-perception in two groups

More than half of the CVD patients (54.1%) and non-CVD patients (57.0%) reported having a normal weight. 35.0 and 8.3% of CVD patients thought they were overweight and obese, compared to 29.5 and 5.8% of non-CVD patients, respectively. Only 2.5% of CVD patients and 7.8% of non-CVD patients thought they were slim. CVD patients and non-CVD patients demonstrated different patterns of self-perceived weight status (***χ***^**2**^ = 8.690, *P* = 0.034).

**Fig. 1:**
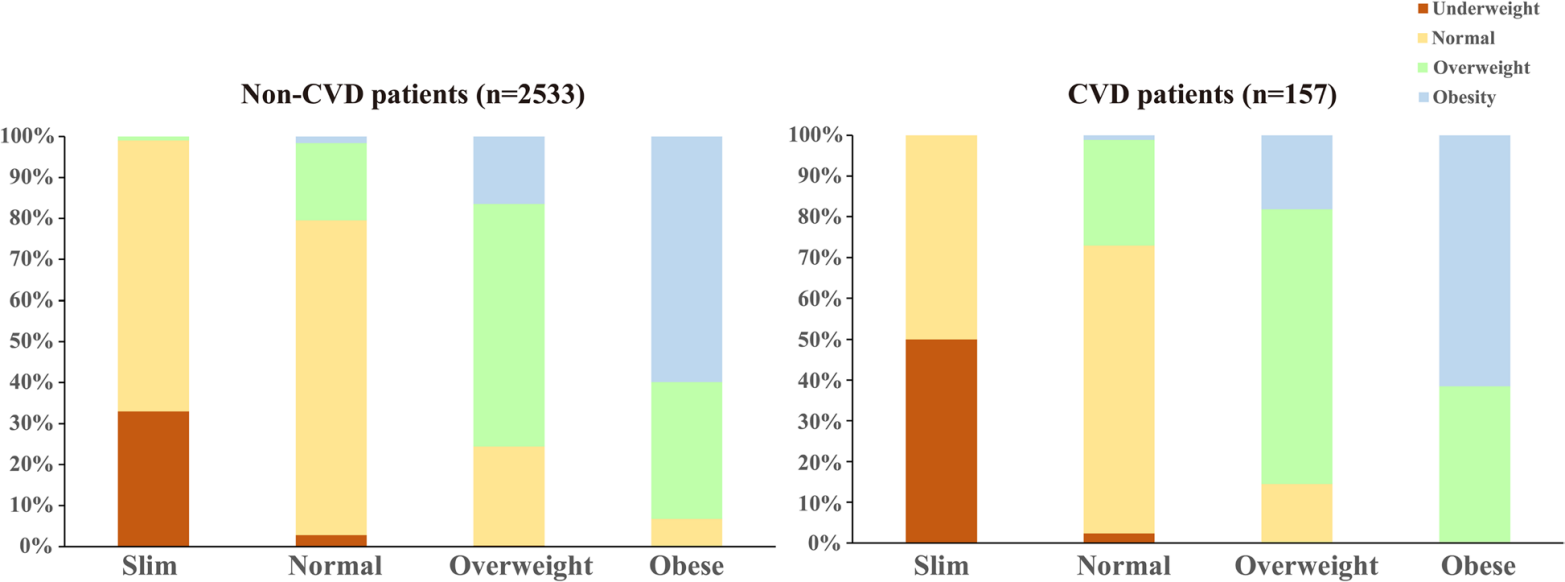
Self-reported weight and actual weight status in non-CVD and CVD patients

For consistency of weight self-perception and actual weight status, over 50% of respondents in the “normal”, “overweight”, and “obese” groups of weight self-perception had a correct self-perception among individuals with or without CVD (Fig. 1). A total of 67.2% of the study population perceived their weight to be in a category that agreed with their actual weight. This translates to a kappa statistic of 0.439. Among non-CVD and CVD patients, percentage agreement and kappa statistics were higher in CVD patients (68.2% and 0.474, respectively) and lower in non-CVD patients (67.2% and 0.436), which indicated higher consistency of self-reported weight and actual weight (Table 2).

**Table 2:** Correct perception, misperception, and kappa statistics of self-reported weight compared to actual weight across groups

	Total ***n*** = 2690	Non-CVD patients ***n*** = 2533	CVD patients ***n*** = 157
Correct perception	1809 (67.2)	1702 (67.2)	107 (68.2)
Misperception	881 (32.8)	831 (32.8)	50 (31.8)
Underestimation (Negative)^a^	585 (21.7)	550 (21.7)	35 (22.3)
Overestimation (Positive)^b^	296 (11.0)	281 (11.1)	15 (9.6)
Kappa^c^	0.439	0.436	0.474

^a^Underestimation means self-reported weight status is lower than the actual weight status

^b^Overestimation means self-reported weight status is higher than the actual weight status

^c^Kappa statistics were estimated to check the consistency between correct perception and misperception separately for CVD and non-CVD patients

### Factors associated with misperception of weight

Misperception of weight was significantly associated with gender, education level, and actual BMI. Females had a greater likelihood to misperceive their weight than males (*OR* = 1.182; 95% *CI*: 1.005-1.338, *P* = 0.042). Misperception of the body weight was more likely to happen in respondents whose education level was less than in secondary school (*OR* = 1.593; 95% *CI*: 1.281-1.980; *P* < 0.001) than in higher education.

According to the multivariate analysis results of factors associated with bodyweight misperception demonstrated (Table 3), people with lower BMI levels were more likely to misperceive their weight than people with normal BMI levels (*OR* = 2.334; 95% *CI*: 1.554-3.505; *P* < 0.001), Overweight and obese people had higher odds ratios of misperceptions compared with normal category, with the highest odds ratio of misperception in the obesity category (*OR* = 5.808, 95% *CI*: 4.361-7.735; *P* < 0.001).

**Table 3:** Relationship between actual body weight and misperception of body weight^a^

BMI groups	Crude Odds Ratio (95% ***CI***)	***P***	Adjusted Odds Ratio (95% ***CI***)	***P***
Normal	Reference		Reference	
Underweight	2.324 (1.559, 3466)	< 0.001	2.334 (1.554, 3.505)	< 0.001
Overweight	2.592 (2.156, 3.116)	< 0.001	2.614 (2.167, 3.154)	< 0.001
Obesity	5.788 (4.367, 7.673)	< 0.001	5.808 (4.361, 7.735)	< 0.001

^a^This model was adjusted for gender and education levels

No significant association was noted between whether suffering from cardiovascular events, respondents’ age groups, marital status, and weight misperceptions in this study.

### Purposes and measures to control weight

**Fig. 2:**
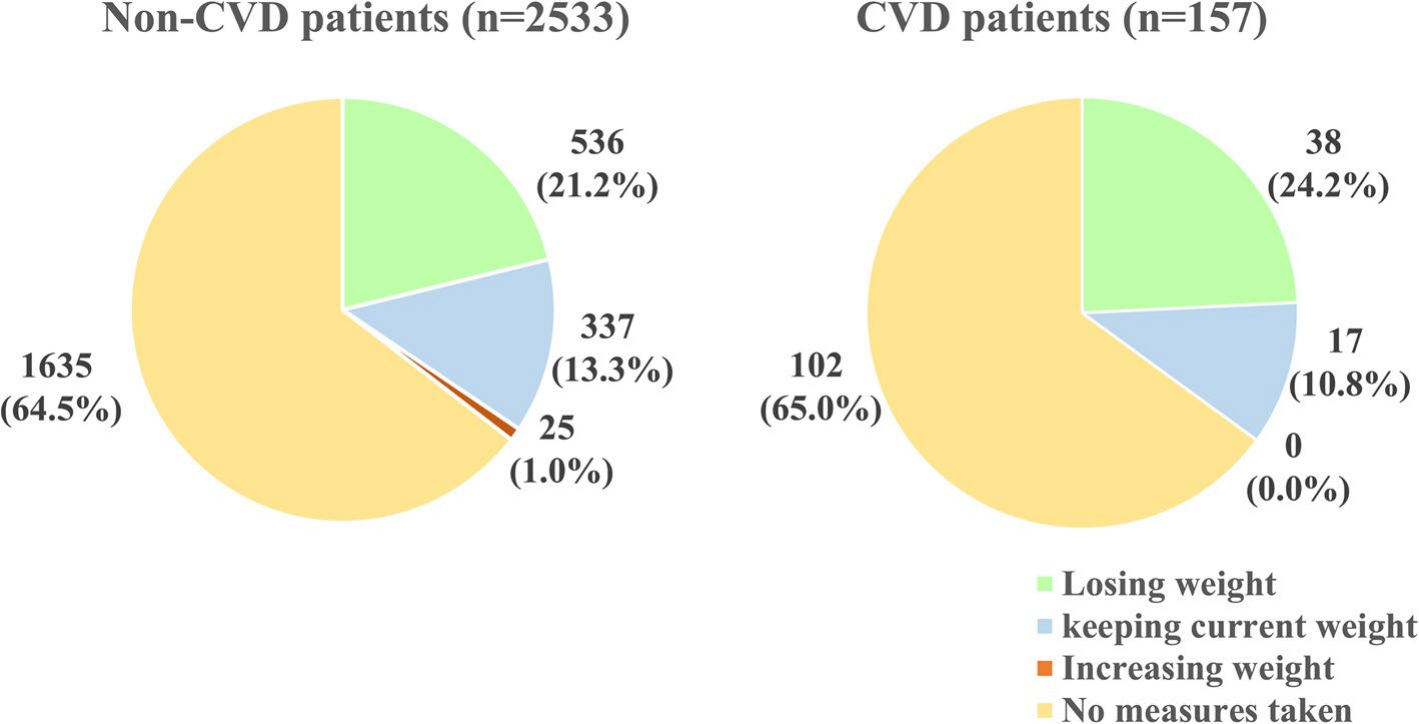
Purpose of taking measures to control weight

When data on purposes and measures to control weight were analyzed, we observed that 64.5% of non-CVD patients and 65.0% of CVD patients didn’t take any measures to control weight. Among respondents who have taken measures to control weight, 21.2% of non-CVD patients and 24.2% of CVD patients were trying to lose weight, while keeping their current weight was another purpose (Fig. 2). Subgroup analyses showed no difference in the purposes of taking measures to control weight in the two groups (***χ***^**2**^ = 2.884, *P* = 0.410). There were 873 non-CVD patients and 55 CVD patients who had taken measures to lose or maintain weight. The most popular measures adopted by the two groups were combining measures of controlling diet and exercise (496 (56.8%) of non-CVD patients and 32 (58.2%) of CVD patients), single approach such as only controlling diet and only exercising was also commonly used.

## Discussion

### Key findings

This study presents four major findings that are novel to the field. First, a higher proportion of 35.0 and 8.3% of CVD patients perceived to be overweight and obese, compared to 29.5 and 2.5% of non-CVD patients. Second, while weight misperception was relatively prevalent among individuals with and without CVD, CVD patients showed a higher consistency between self-reported weight and actual weight than non-CVD patients. Third, misperception of weight status was more likely to happen among obese respondents, women, and individuals with lower education levels. Last, no difference was found in the purposes of taking measures to control weight among CVD or non-CVD patients. The most adopted weight loss measures in respondents who wanted to lose or maintain weight were combined measures including controlling diet and exercise.

### Interpretation of findings

In this study, we disclosed that weight misperception was significantly associated with actual BMI, education level, and gender in Chinese adult populations. BMI is an important factor associated with self-weight perception. In accordance with other research among adults in Saudi Arabian, Malaysia, and adolescents in Wuhan, China [17–19], our findings indicated that obese populations were more likely to misperceive their weight than respondents with normal BMI levels. This indicates that obese populations may generate a new criterion for weight status besides the actual standard, while obesity is considered as “overweight” or even “normal” in their opinion, which made a vicious circle and a barrier to maintaining weight that should be a social concern.

Also, our study demonstrated participants with lower education levels are particularly vulnerable to weight misperception, in line with Norfolk and older Dutch adults studies [20, 21]. Furthermore, an ECHORN cohort study for Eastern Caribbean adults also found that participants with higher education levels versus lower education levels had decreased 50% odds of weight misperception [22]. The above conclusions suggest that implementing health education and improving cognitive levels can effectively avoid weight misperception. The results also revealed that males were less likely to misperceive their weight than females [17, 23]. In the internet age, females are more likely to be influenced by appearance and weight anxiety from the internet and persuaded to lose weight though they are normal even underweight. Therefore, the healthy weight concept should be proposed to avoid unnecessary anxiety about weight, and healthy habits and diet should be promoted to maintain an appropriate weight.

In this study, the multiple variable analysis depicted that CVD prevalence is not associated with weight misconception, although CVD patients had a higher proportion of overweight and obese in our findings. As we know, weight management and health education are important measures in the primary and secondary prevention of CVD [24]. And having correct weight self-perception is a vital part of health education about weight control. Therefore, our results indicated the progress in health management and intervention for CVD patients on weight perception, which was consistent with non-CVD patients and avoiding weight misconception. Since weight perception is highly related to weight loss attempts [10, 25], overweight and obese states in CVD patients are expected to improve in the future.

The relatively low percentage of respondents who took measures to lose or keep weight reveals the urgency of increasing awareness of weight control, especially for individuals with obesity-related chronic diseases [26, 27]. Healthy education should be done to guide people to keep weight at a healthy level without weight anxiety. Encouragingly, more than half of the respondents who had taken measures to lose or maintain weight adopted combined measures including controlling diet and exercise. This reflects considerable progress in proposing and implementing a healthy weight loss program, since combined diet and physical activity have been confirmed that benefit more than diet or exercise alone in improving health outcomes [28, 29]. Continued work is needed for individual treatment including a weight loss program for CVD patients according to underlying disease status and physical condition [30].

### Strengths and limitations

Our findings have three major implications. First, achieving and maintaining a healthy weight is one of the primary prevention for CVD, however, weight misperception is a barrier to maintaining weight [30, 31]. Our work took the lead in studying the weight self-perception and misperception status in CVD patients, which is a bellwether for subsequent studies concerning weight misperception in primary prevention and integrative treatment of CVD. Second, we analyzed the association between sociodemographic factors and the occurrence of weight misperception in the Chinese adult population for the first time, which refined the target groups for health promotion and education. Third, based on the close connection between weight perception and weight attempt, we also draw a picture of weight attempt and weight loss strategies taken by individuals with and without CVD, encouraging more effective guidance of precise individual weight loss plans.

This study is primarily limited by its use of the China HeartRescue Global Evaluation Baseline Household Survey 2015, which is a retrospective cross-sectional study. Moreover, the sample is limited to Beijing and Shanghai and may not represent all Chinese population. Finally, a small sample size of CVD patients, no access to the incidence of CVD, and limited risk factors prevented further and more accurate analysis of weight self-perception and CVD, which could be substantially overcome through the inclusion of high-quality raw data in future research.

## Conclusions

Our results indicated that weight misperception was relatively prevalent both in individuals with and without CVD. A higher consistency between self-reported weight and actual weight was noticed in CVD patients. Furthermore, misperception of weight status was more likely to happen among obese respondents, women, and individuals with lower education levels. Particular attention and work should be put into understanding and education with these populations because of their vulnerability.

## Data Availability

The data that support the findings of this study are available from China HeartRescue Global Evaluation Baseline Household Survey 2015, which is an open access resource. Bona fide researchers can apply to use relevant data provided by Institute for Health Metrics and Evaluation (IHME) at Global Health Data Exchange: http://ghdx.healthdata.org/record/ihme-data/china-heartrescue-global-evaluation-baseline-household-survey-2015.
